# Study on the impact of digital transformation on green competitive advantage: The role of green innovation and government regulation

**DOI:** 10.1371/journal.pone.0306603

**Published:** 2024-08-01

**Authors:** Shaoling Wu, Peng Cheng, Fan Yang

**Affiliations:** 1 School of Economics and Management, Hubei University of Automotive Technology, Shiyan, Hubei, China; 2 Dongfeng Commercial Vehicle Co., Ltd, After-Market Business Division, Shiyan, Hubei, China; 3 School of Foreign Languages, Hubei University of Automotive Technology, Shiyan, Hubei, China; East China Normal University, CHINA

## Abstract

Digital transformation enables small and medium enterprises (SMEs) to reduce or overcome their reliance on resources and energy, thereby minimizing their environmental impact and providing them with sustainable green competitive advantages. However, the reasons for this phenomenon are not yet clear. To further investigate this issue, we selected 391 Chinese SMEs to examine the relationships among green transformation, green innovation, government regulation, and green competitive advantages. Green innovation includes green product innovation and green process innovation, while government regulation includes incentive regulation, constraint regulation, and guidance regulation. The empirical results show that digital transformation can enhance SMEs’ green competitive advantages. Additionally, the hypothesized mediating effect of green product innovation and green process innovation between digital transformation and green competitive advantages is supported, while the moderating effect of incentive regulation, constraint regulation, and guidance regulation on the relationship between digital transformation and green product innovation and green process innovation is also confirmed. The findings of this study may contribute to more effective management of digital transformation and green innovation in SMEs, thereby promoting their development.

## 1. Introduction

The rapid economic development, while addressing people’s material needs, has also led to significant resource wastage and environmental degradation, resulting in a series of ecological conflicts and economic issues such as air pollution, water contamination, and food safety concerns. In the increasingly complex global economic landscape, heightened environmental awareness, the burgeoning green economy, governmental prioritization of environmental issues, and the strengthening of green trade barriers on the international market are compelling SMEs. As principal actors in the market economy, to face substantial challenges in building green competitive advantages to meet market demands and societal expectations. Additionally, the ongoing development of the big data sector and digital transformation and green innovation play a crucial role in production activities, which is particularly emphasised in industry, which has been recognized by the Chinese government. Since January 2023, the Chinese government has initiated comprehensive pilot projects in ten regions, including Zhangjiakou in Hebei province and Dalian in Liaoning province, to strongly advocate the deep integration of digital technology and green concepts, low-carbon industries. These initiatives demonstrate China’s significant emphasis e-growth and eco-growth together in pursuit of high-quality economic growth. Therefore, against the backdrop of global green transformation, the universality and inevitability of digital transformation practices have sparked sustained interest in academia and industry. The potential of digital transformation to enable SMEs to achieve green development goals, thereby helping them establish a green competitive edge, has become a critical area of focus. Exploring technological and managerial strategies to enhance the green competitive advantage of SMEs has emerged as an urgent topic for research by governments and businesses alike.

Although transformation of businesses in terms of digitalisation and competing interests in terms of environmental protection have gradually received attention for the last few years, most scholars have conducted separate studies on the two, rarely combining them [1]. Research on digital transformation is still in its early stages, with current studies focusing more on the digital transformation of enterprises themselves, including the application of digital technologies to enhance market performance [2] financing constraints [3], among other aspects. Existing studies have explored the factors that enhance green competitive advantage in depth [4], but there is inadequacy of information on the pathways through which digital switching digital tr ansformation affects green competitive advantage. Therefore, there is a theoretical gap in understanding how enterprises can effectively achieve the ombination of digital conversion and green competitive advantage. Empirical discussions about the connection between digital transformation and sustainability and green competitive advantage are increasing [5], yet the results remain uncertain. The lack of a basic theoretical framework is considered an obstacle to further understanding the synergy between digital modernization and environmental sustainability competitive advantage [6]. A small number of scholars have begun to focus on the correlation between corporate digital advancement and environmental sustainability competitive advantage, and recent studies have indicated that the extent to which digital transformation ultimately translates into green competitive advantage may be influenced by green innovation and government regulations [7]. However, these studies have remained sporadic and superficial in their theoretical analyses [8], with a scarcity of research that takes both as the main topic and conducts in-depth analysis of the mechanisms between them. In fact, in the context of vigorously implementing the innovation-driven development strategy and green transformation, how to utilize digital transformation as an important measure for SMEs to develop a competitive position of ecological advantage has become increasingly significant [9].

With the rapid development of digital technologies such as intelligent algorithms and massive data, data-based transformation is considered to play a crucial role as an innovation "engine" [10]. This is mainly because digital transformation enhances the ability to search, organize, and transform data to uncover critical knowledge information that supports enterprise decision-making and production [11]. It not only permeates various levels of business through cutting-edge and integrated technologies but also stimulates intelligent responses to customers and enhances collaboration efficiency between different units, nurturing sustainable competitiveness. Digital transformation can also shaping the competitive position of eco-advantage through the importance of environmental innovation. SMEs can leverage digitalization to access external knowledge, especially dynamic information on energy conservation, emission reduction, and environmental governance [12], further promoting green innovation among these enterprises. Green innovation emphasizes the harmonious development of the economy, society, and environment, bringing intangible assets to SMEs, including corporate Public Image, green reputation, and strengthened competitive position [13]. Therefore, in the context of the green economy,new developed and implemented products and processes need to demonstrate more environmentally friendly characteristics than ever before [14], with more organizations actively adopting green innovation to achieve sustainable competitive development in an ecologically effective manner [15], strengthening the ecological competitive position of enterprises [16]. As significant promoters of digital change and innovation in environmental concepts, local governments play a fundamental and guiding role in helping SMEs build green competitive advantages. Their formulation of incentive or regulatory policies (i.e., government regulations) can influence the behavior of all small and medium-sized business entities under their management. Existing research findings have shown that government regulations play a crucial role in advancing the company’s digitalisation and eco-innovation. Research needs to explore the specific impact of government regulations on digital Transformation and Eco-Innovation in enterprises, particularly in clarifying the mechanisms through which government regulations affect digital transformation and utilise eco-innovation to enhance business competitiveness in the green sector [17]. Eco-friendly competitive edge, as one of the key factors for SMEs to achieve durable development, is a long-term competitive advantage that will not disappear with environmental changes. It helps SMEs avoid strategic replication or imitation by potential competitors, enabling them to gain long-term benefits. Additionally, green competitive advantage helps SMEs reduce production costs, improve product quality, and enhance overall performance. Furthermore, with the widespread adoption of green consumption, the demand for green products continues to grow. Opportunities for SMEs to increase their share of the market and develop more green products and services that meet market demands by building green competitive advantages, thus helping shape a positive brand image, enhance brand value, and increase brand awareness. The future strategic and competitive advantage lies in the ability to promote green sustainable economic activities, and wise enterprises will choose strategies related to green initiatives to shape green competitive advantages [18].

Dynamic capabilities play a positive role in enhancing the competitiveness of SMEs by enabling them to adapt to rapidly changing environments and make dynamic adjustments [19]. They also help SMEs effectively respond to strategic and operational changes, enhancing operational capabilities. Dynamic capabilities are considered the ability of organizations to purposefully create, expand, and adjust their competitive advantages. When scaling up, SMEs developing dynamic capabilities is essential for innovation and maintain a competitive advantage relative to other enterprises [20]. Research has found that dynamic capabilities play a central role in the digital transformation process of SMEs [21]. Digital transformation is not simply the digitization of existing capabilities; SMEs need to leverage their existing capabilities while continuously pursuing the discovery of new capabilities in business processes. By using sensing capabilities to identify potential opportunities and challenges from within and outside the organisation, SMEs can maintain flexible capacity for innovation potential and competitive advantage in the market. To acquire and maintain of dynamic capabilities, SME managers need to stay sharp and observant and innovative awareness [22]. By innovatively transforming resource bases and developing cutting-edge knowledge-intensive products, SMEs can increase green innovation output to sustain their competitive advantage [23]. Therefore, this study investigates how SMEs can enhance their green competitive advantage through looking at digital transformation through the lens of dynamic capabilities. It focus on research that provides insights into the mechanisms and outcomes of how digital transformation affects the green competitive advantage in SMEs. The study incorporates introduce green process innovation into the research architecture and exploring the articulation function of green innovation in digital transformation relevance and green competitive advantage in SMEs. Furthermore, based on the perspective of government regulations, the study explores the boundary effects of government regulations on the relationship between digital transformation and green innovation.Based on the previous research, this study is subdivided into three sub-questions for ease of interpretation:(1) What is the influence of digital transformation on the green competitive advantage of SMEs? What are the pathways of their effects? In response to the increasingly complex and volatile market conditions, many SMEs have either already begun or are preparing to undergo digital transformation. Some enterprises have even made significant progress in this regard. However, their individual experiences are not sufficient to cover the entire industry, and some enterprises still maintain a wait-and-see attitude toward digital transformation. Therefore, the first sub-question regarding this investigation aims to investigate the impact and pathways of digital transformation on the green competitive advantage of SMEs, in order to clarify the significance of digital transformation for enterprises and provide theoretical guidance for their digital transformation. (2) What role does green innovation play for SMEs during the process of digital transformation? In the previous research, many scholars have empirically studied the correlation between green innovation and a firm’s green competitive advantage using various methods. However, their conclusions are not entirely consistent, indicating that the relationship between green innovation and a firm’s green competitive advantage is extremely complex. This complexity arises from the multidimensional nature of green innovation, and the quality of a firm’s green competitive advantage depends on different dimensions of green innovation capability for firms of different sizes. Therefore, the second sub-question of this study, within the new perspective of digitalization, aims to explore the role of green innovation in the relationship between digital transformation and a firm’s green competitive advantage. (3) How does government regulation regulate the relationship between digital transformation and green innovation for SMEs? In today’s increasingly fierce market competition, global economic growth slowdown, geopolitical conflicts, and other factors have brought significant uncertainty to the development of world trade. Government regulation is particularly important for business operations, as it represents an effective choice for coordinating economic sustainability and green low-carbon development. Through regulatory measures, the government can either compel, incentivize, or guide enterprises to undergo digital transformation and develop in a green manner. However, there is little literature that introduces government regulation as a variable in the study of digital transformation and green development. Therefore, the third sub-question of this study is to consider government regulation as a moderating variable and explore its moderating role in the relationship between digital transformation and green innovation in SMEs, in order to reveal the boundary conditions of digital transformation on green innovation.

In comparison with previous studies, this paper’s marginal contributions mainly lie in the following aspects: Firstly, existing research has not deeply analyzed the mechanisms through which digital transformation influences a firm’s green competitive advantage. This paper, based on the perspectives of internal green innovation and external government regulation, clarifies the potential mechanisms of digital transformation on SMEs’ green competitive advantage, thus contributing to the expansion of the theoretical research boundaries of existing green competitive advantage. Secondly, based on the structured hierarchical dimensions of green innovation, this paper explores the differentiated effects of green product innovation and green process innovation on a firm’s green competitive advantage. It also analyzes the heterogeneous effects of different dimensions of green innovation on green competitive advantage, enriching the literature on the dimensions of green innovation and green competitive advantage. Thirdly, current scholarly research emphasizes the correlation between digital transformation and a firm’s economic value, while research on the relationship between digital transformation and non-economic values such as a firm’s green competitive advantage is relatively scarce. This paper analyzes the specific impact of digital transformation on SMEs’ green competitive advantage from a micro perspective, thus supplementing the academic research on the relationship between digital transformation and green competitive advantage. Lastly, existing dynamic capability theory may overlook the importance of long-term green competitive advantage while focusing on the short-term performance of medium and small enterprises. In the process of digital transformation, medium and small enterprises need to pay more attention to the development of green, low-carbon, and environmentally friendly aspects to achieve a mutually beneficial outcome in terms of economic and social benefits. Therefore, integrating the concepts of digitalization and green innovation into dynamic capability theory to form a dynamic capability view of digitalization and greenization can enrich dynamic capability theory, allowing the dynamic capabilities of medium and small enterprises to reflect the characteristics of digitalization and greenization.

## 2. Research hypothesis

### 2.1 Direct effect of digital transformation on green innovation relationship

Digital transformation is considered a new engine for promoting socio-economic development, while green innovation is seen as a key measure for developing green practices. Therefore, digital transformation should ’give back’ to green innovation to achieve the harmonious unity of economic, social, and ecological environments. Existing research shows that digital transformation in small and medium-sized businesses have a positive effect on environmental innovation [24]. Scholars typically categorize green innovation is divided into two categories: based on environmental process innovation and environmental product innovation different theoretical backgrounds and research perspectives [25]. Process innovation in the green sector involves innovating operational systems and management processes to meet environmental protection requirements, for example, energy efficiency, pollution control and waste recycling, or non-toxic practices. Meanwhile, environmental product innovation focuses on environmental protection aspects throughout the product lifecycle, covers energy saving, pollution management, waste reuse and environmental product design [26]. Specifically, SMEs can integrate digital technologies into their production processes [27], enhance internal information sharing and processing capabilities, facilitate cross-departmental collaboration and coordination, and empower business practices through digital transformation to improve business flexibility [28]. Moreover, digital transformation can optimise resource allocation and develop competitive new products by combining digital technology with existing products [29], integrate green supply chains, and facilitating business processes in the supply and distribution chain, thereby promoting innovation in environmentally friendly products in SMEs [30]. Therefore, digital transformation permeates various aspects of innovation in the field of environmental protection and innovation in environmental protection technology, effectively driving environmental protection innovation in SMEs. Based on this, the following hypotheses are proposed:

H1: Digital transformation is positively related to green product innovation (a) and green process innovation (b).

### 2.2 Direct effect of digital transformation on the relationship with green competitive advantage

To cope with increasingly fierce market competition, many enterprises are proactively or involuntarily undergoing digital transformation, with digital strategies gradually becoming the core pillar of enterprise strategies, building competitive advantage in the marketplace over the long term. Martínez-Caro et al. emphasized the necessity of digital technology in achieving competitive advantages and concluded that SMEs should focus on digital technologies, including computing, information integration, and connectivity technologies, to achieve sustainable competitiveness [31]. Furthermore, these digital technologies play a crucial role in enterprise strategic development, as digital transformation can help companies create green competitive advantages [32]. Empirical studies by Kwon et al. have shown that applying advanced digital technologies, particularly big data technologies, in the supply chain can improve data management quality and enhance market competitiveness effectively [33]. Additionally, Verhoef et al. mentioned that the purpose of transitioning to digital technology is to provide more value to enterprises while also creating green competitive advantages [34]. Xue et al. pointed out that to gain green competitive advantages, organizations must change the original logic of their services and drive digital transformation in all environments, including operations, structures, and strategic planning [35]. Schwertner suggested that companies viewing technologies such as cloud, big data, social, and mobile as essential parts of their infrastructure compared to competitors would be more profitable and have higher market valuations [36]. Therefore, these technologies serve as key drivers and tools for obtaining green competitive advantages through digital transformation. Therefore, we propose the following hypothesis:

H2: Digital transformation is positively related to green competitive advantage.

### 2.3 Direct effect of green innovation on the relationship with green competitive advantage

With the increasing global focus on environmental sustainability, environmental innovation has become an important strategy approach for companies to maintain competitiveness and meet consumer demands. Green innovation, as an intangible strategic capability, possesses value, rarity, inimitability, and non-substitutability. Particularly, implementing innovative measures for environmental protection strategies is crucial for SMEs to establish a advantage in competition. Through continuous investment in green capital and research and development, SMEs can enhance the greenness of their products, processes, and end-of-life management, achieving a full lifecycle greening of production and sales to address pollution issues and establish a competitive edge [37]. Specifically, throughout the product’s life cycle, Green Innovations products endeavour to reduce dependence on resources and energy., reduce the use of toxic materials when designing products, increase the useful life of older products, or create recycling programmes [38]. Leading companies implementing through the sale of environmentally friendly technologies and the provision of services, green and innovative products not only enhance a company’s brand image, but may also open up new market segments, thereby gaining a competitive advantage in environmental protection [39]. Green process innovation, on the other hand, emphasizes that in the face of continued rising demand for green consumption, companies differentiate themselves from competitors by lowering costs, enjoying a competitive advantage, and establishing a green brand image, gaining consumer trust, and ultimately more competitive in the marketplace [40]. Therefore,green innovations in products and processes can effectively reduce production costs and increase production efficiency while optimising product quality, allowing SMEs to gain a competitive edge through the implementation of environmental innovation [18]. Based on this, the following hypotheses are proposed:

H3: Green product innovation (a) and green process innovation (b) are positively related to green competitive advantage.

### 2.4 The mediating role of green innovation in the relationship between digital transformation and green competitive advantage

Digital transformation integrates digital technologies with business processes, helping SMEs innovate and upgrade existing technologies, products, and business processes to enhance their market competitiveness [41]. Empirical studies have shown that an increase in digitalization levels effectively enhances technological integration capabilities, thus stimulating green innovation in SMEs [8]. green innovation, as a general skill for SMEs, not only has cost reduction and quality improvement advantages at the product and production process levels but also drives the emergence of products with performance and brand advantages and green production lines, creating new products that excel in performance, match market personalized and specialized demands, and reconstruct product market competitiveness [42]. Digital transformation, according to Xie, optimizes process flows to green the production process [43], efficiently allocates resources, facilitates the formation of multi-party participation in green innovation network organizations, and enhances green process innovation to bring greater green competitive advantages to enterprises [26]. Environmental product innovation can increase the greenness of products, create differentiated products, reduce costs, establish green brand images, and achieve a full lifecycle greening, providing multiple green competitive advantages for enterprises [37]. It is evident that green process innovation and green product innovation serve as a bridge between digital transformation and competitive position with ecological advantages. Therefore,we propose the following hypothesis:

H4: Green product innovation (a) and green process innovation (b) positively mediate the relationship between digital transformation and green competitive advantage.

### 2.5 The regulatory role of government regulation in the relationship between digital transformation and green innovation

In the context of sustainable development, a series of government regulations that emphasize green innovation have been introduced, playing an important role in improving innovation in the green sector. in SMEs. Government regulation refers to the government’s establishment of relevant systems through administrative, economic, and social means to fulfill its functions of incentive and constraint, guiding and regulating business behavior, and adjusting economic behavior through regulatory measures to harmonize the relationship between economic development and ecology. It can be divided into restrictive regulation, incentive regulation, and guiding regulation [44]. The process of digitizing operations and embracing technology.is becoming a key driver for the process of digitizing operations and embracing technolog for SMEs, the impact of the digital shift on environmental process innovation and technological innovation is regulated by government regulations [45]. Firstly, based on the formulation of environmental laws and regulations, the government strengthens law enforcement and imposes penalties on violations. After weighing the costs of non-compliance, SMEs plan to use digitally transformative technologies such as big data and artificial intelligence to reduce economic and environmental costs, thereby enhancing their green innovation capabilities [46]. Secondly, the government adopts incentive measures such as credit support, financial subsidies, and tax reductions to facilitate the commitment of enterprises to the reform of digital switchover, reduce the costs of green entrepreneurship opportunities for SMEs, and promote stable revenue expectations, thereby enhancing innovative solutions for a sustainable environment in SMEs [47]. Lastly, through environmental education, technology that is eco-friendly and sustainable is referblue to as green technology training, and the establishment of innovative solutions for a sustainable environment models, the government can enhance environmental awareness and green development concepts in SMEs, prompting them to introduce digital technologies in aspects such as production, management, and research and development, thereby enhancing their green innovation capabilities [48]. Therefore, under the regulation of government regulations, digital change helps companies achieve high levels of green innovation, especially playing a more significant role in promoting green innovation in SMEs. Given this information, the following hypotheses are suggested:

H5-1: Incentive regulation positively moderates the relationship between digital transformation and green product innovation (a) and green process innovation (b).
H5-2: Constraint regulation positively moderates the relationship between digital transformation and green product innovation (a) and green process innovation (b).
H5-3: Guiding regulation positively moderates the relationship between digital transformation and green product innovation (a) and green process innovation (b).

**Fig 1:**
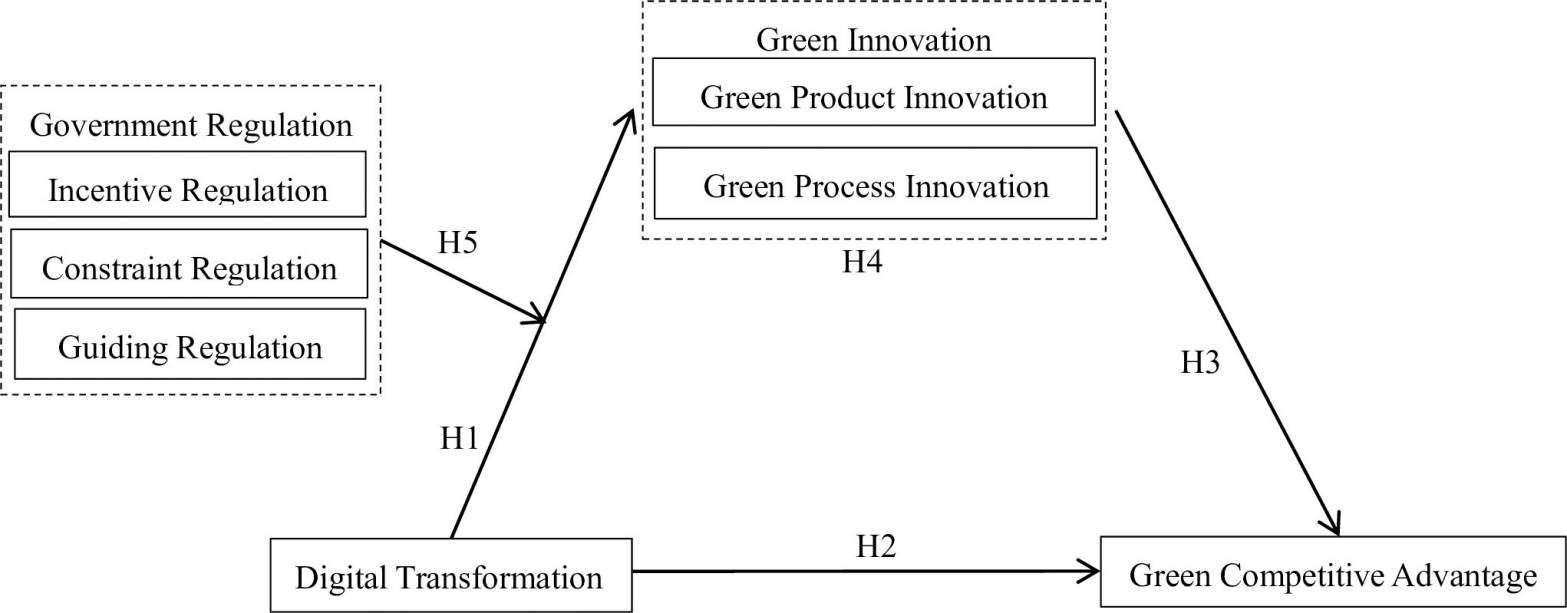
Research framework.

Fig 1 illustrates investigate framework of this article.

## 3. Research approach

### 3.1 Data gathering and sampling

This study adopts a quantitative method to investigate the relationship between green innovation, government regulation, and green competitive advantage in businesses. Given that the data used in this study are original data collected to enrich the literature on SMEs’ management, we conducted a survey study using a questionnaire. Based on the research object of Chinese SMEs, this study selects the standard for dividing Chinese enterprises into four types of large-, medium-, small-, and micro-types, which refers to the documents "Circular on Issuing the Regulations on SMEs’ Type Standards" (No. 300 of the Ministry of Industry and Information Technology and the National Industry and Information Technology [2011]) and "National Economic Industry Classification" (GB/T4754-2017) and adopts snowball sampling through alumni associations and entrepreneurs associations. Before administering the survey instrument, data was collected via freely flowing, systematic interviews, and cell phone interviews. The interview questions included basic information about the interviewed information through freely flowing, structured interviews, and telephone companies, current production and operation, digital technology, green quiz innovation activities, sustainable competitive advantages, and government green policies. Based on the reactions received from these interviews, we supplementary refined the survey. In addition, we extended 5 professors specialized in business management to critically assess questionnaire content, and evaluate made modifications in accordance with feedback. After initial inquiry was designed, we administered a pre-survey with 15 companies, and made modifications to some of the questions in the initial questionnaire based on their feedback and suggestions. Through these measures, we formed the final survey questionnaire. The ultimate survey quiz is broken down into two components, centering on the digital transformation, green innovation, and green competitive advantage of enterprises. The first part outlines the basic information and characteristics of the enterprises. The second part mainly discusses the digital transformation and green innovation of the enterprises.

### 3.2 Construct measurements

The survey questionnaire utilized the Likert seven-point measurement scale, spanning (1) "strongly disagree" (7) "strongly agree." For measurement of digital transformation, a scale proposed by Nasiri et al. was used [49], consisting of 8 items. For the measurement of green innovation, the research findings of Chen et al. and SHU et al. were employed [26, 50]. Specifically, green product innovation incorporated 4 measurement components, and green process innovation also comprised 4 measurement items. The assessment of environmental impact, green competitive advantage was derived from the measurement instrument created by Leonidou et al. [51], consisting of 6 measurement items. For the measurement of government regulation, the research findings of Yu et al. were utilized [52]. In this context, incentive regulation comprised 3 measurement items, constraint regulation comprised 4 measurement items, and guidance regulation comprised 3 measurement items. Generally, digital transformation, green innovation, and others are affected by the basic attributes of enterprises [53]. This study employed control variables to ensure accurate research results [54], considering company age, number of employees, annual income, and industry.

### 3.3 Demographical distribution analysis

The survey was conducted between November 2022 and June 2023. Prior to the initiation of the study, the purpose and procedure of the research were elucidated to all participants, alongside their rights, accompanied by the presentation of an informed consent form. Upon securing their verbal informed consent, participants proceeded to complete the questionnaire. and privacy of all participants was guaranteed, participants’ confidentiality was maintained, with the freedom to withdraw from participation at any point. In total 500 survey forms were distributed, and after excluding incomplete or patterned responses, survey generated an impressive response rate of 78.2%, with 391 legitimate opinion polls successfully collected. The specimen was drawn from 391 SMEs across 20 provinces in China. In terms of company age, there were 128 enterprises with less than 5 years of operation, 126 with 5–10 years, and 137 with over 10 years. Regarding the number of employees, there were 118 enterprises with fewer than 50 employees, 130 with 50–300 employees, 94 with 300–1000 employees, and 49 with over 1000 employees. In terms of annual income, there were 104 enterprises with less than 20 million RMB, 127 with 20–500 million RMB, and 160 with over 500 million RMB. In terms of industry, 77 enterprises were in the agricultural sector, 65 in the service sector, and 249 in the industrial sector.

## 4. Data analysis and results

### 4.1 Reliability and validity analysis

According to the research hypothesis, digital transformation has a positive impact on the green competitive advantage of SMEs, with green innovation as the mediator, and government regulation as the moderator for impact of digital revolution centered around sustainable innovation. Table 1 showcases the implications arising from the structural validity as well as reliability assessment conducted with SPSS v.26.0.

**Table 1:** Constructs and measures.

VARIABLES	ITEMS	Cronbach’s α	FACTOR LOADING	AVE	CR
Digital transformation	In my organization, our goal is to digitize everything that can be digitized.	0.895	0.770	0.636	0.897
	In my organization, we have collected a large amount of data from various sources.		0.800		
	In my organization, our goal is to leverage digital technology to create a more robust network between different business processes.		0.801		
	In my organization, our goal is to enhance efficient customer interfaces through digitization.		0.787		
	In my organization, our goal is to achieve digitalization for information exchange.		0.829		
Green product innovation	When developing or designing products, we choose materials that generate the least amount of pollution.	0.893	0.800	0.677	0.893
	When developing or designing products, we choose materials that consume the least amount of energy and resources.		0.853		
	When developing or designing products, we aim to use the minimum amount of materials necessary to construct the product.		0.814		
	When developing or designing products, careful consideration is given to whether the product is easily recyclable, reusable, and decomposable.		0.822		
Green process innovation	This company’s manufacturing process effectively reduces the emission of hazardous substances or waste.	0.871	0.774	0.634	0.874
	This company’s manufacturing process involves the recycling of waste and emissions, ensuring they are treated and reused.		0.810		
	This company’s manufacturing process reduces the consumption of water, electricity, coal, or petroleum.		0.820		
	This company’s manufacturing process reduces the use of raw materials.		0.780		
Green competitive advantage	A company with environmental awareness can bring cost advantages.	0.919	0.810	0.657	0.920
	By striving to improve environmental quality, we have achieved significant cost advantages.		0.841		
	By investing in the development of environmentally friendly products, our company can become a market leader.		0.828		
	Our company can enter profitable new markets by adopting an environmental strategy.		0.786		
	Our company can penetrate the market by making existing products more environmentally friendly.		0.789		
	By reducing the environmental impact of our business activities, the quality of our products will improve.		0.809		
Incentive regulation	Implementing environmental protection measures can lead to government funding support or tax incentives for the company.	0.910	0.882	0.772	0.911
	The government provides financial support to companies that implement clean production or initiatives beneficial to environmental improvement.		0.896		
	The government highly values the recognition and promotion of environmentally friendly production enterprises.		0.858		
Constraint regulation	The government has formulated relatively complete environmental regulations and standards.	0.911	0.855	0.721	0.912
	The environmental protection department is very strict in environmental regulation.		0.880		
	The possibility of detection of enterprises violating environmental regulations is very high		0.861		
	Government departments will impose severe sanctions on enterprises that do not comply with environmental management		0.799		
Guiding regulation	The government has made great efforts to promote green agricultural production technology	0.885	0.890	0.730	0.890
	The government has made great efforts in promoting its policies to support agricultural green development.		0.790		
	The government has made great efforts in publicity and education on agricultural environmental protection and governance		0.879		

Table 2 illustrates visible consequences of CFA. Model 1 illustrates a nonpayment model, assuming no interdependence among the indicators In Model 2, green product innovation as well as green process innovation are aggregated into single factor, concurrently digital transformation, green competitive advantage, incentive regulation, constraint regulation, and guidance regulation are amalgamated into a distinct factor. Model 3 constitutes a three-factor model merges green product innovation and green process innovation, digital transformation and green competitive advantage, as well as incentive regulation, constraint regulation, and guidance regulation as three separate factors. Model 4 consists of four factors, namely green product innovation and green process innovation, digital transformation, and incentive regulation, constraint regulation, and guidance regulation. Model 5 includes five factors: green product innovation, green process innovation, digital transformation, green competitive advantage, and incentive regulation, constraint regulation, and guidance regulation. Based on the six-factor model, Model 6 comprises digital transformation, green product innovation, green process innovation, green competitive advantage, guidance regulation, incentive regulation, and constraint regulation. Model 7 treats digital transformation, green product innovation, green process innovation, green competitive advantage, guidance regulation, incentive regulation, and constraint regulation as seven separate factors. Table 2 also indicates that the seven-factor model better fits the observed data than the alternative models (χ2 = 443.092, df = 356, χ2/df = 1.245, RMR = 0.035, GFI = 0.930, NFI = 0.951, IFI = 0.990, CFI = 0.990, RMSEA = 0.0025).

**Table 2:** Comparison of the measurement models for the main variables.

Model	χ^2^	df	χ^2^/df	RMR	GFI	NFI	IFI	CFI	RMSEA
Model 1	3822.621	377	10.14	0.144	0.387	0.580	0.605	0.604	0.153
Model 2	2772.458	376	7.374	0.157	0.491	0.696	0.671	0.725	0.128
Model 3	1308.664	374	3.499	0.071	0.751	0.856	0.893	0.893	0.080
Model 4	777.584	371	2.096	0.046	0.868	0.915	0.953	0.953	0.053
Model 5	706.956	367	1.926	0.044	0.879	0.922	0.910	0.961	0.049
Model 6	634.827	362	1.754	0.042	0.888	0.930	0.965	0.969	0.044
Model 7	443.092	356	1.245	0.035	0.930	0.951	0.990	0.990	0.025

The mean values of the data points main measured parameters are shown in Table 3, ranging from 4.928 to 5.191. Pearson correlation coefficients indicate positive correlations between the factors. Moreover, the main matrix diagonal shows the square root of a number mean variance in statistics extracted (AVE) numerical values, all of which are greater than the inter-construct correlations. These findings indicate that the framework fit is capable of assessed utilizing analysis of regression techniques.

**Table 3:** Descriptive statistics of the measured variables.

	MEAN	SD	SQUARED CORRELATION COEFFICIENTS						
			1	2	3	4	5	6	7
Digital transformation**(1)**	5.017	1.003	**0.798**						
Green product innovation**(2)**	5.187	0.700	0.570**	**0.822**					
Green process innovation**(3)**	5.191	0.757	0.651**	0.806**	**0.796**				
Green competitive advantage**(4)**	4.928	0.913	0.608**	0.534**	0.608**	**0.811**			
Incentive regulation**(5)**	5.135	1.010	0.359**	0.373**	0.382**	0.429**	**0.879**		
Constraint regulation**(6)**	5.133	0.972	0.455**	0.459**	0.470**	0.550**	0.768**	**0.849**	
Guiding regulation**(7)**	5.185	0.982	0.356**	0.351**	0.362**	0.461**	0.767**	0.815**	**0.854**

Note

** means significant at 1% level

* means significance at 5% level.

### 4.2 Hypotheses testing results

Table 4 presents the results with green product innovation as the response variable, Models 1–8 show no multicollinearity (AVIF < 5). Model 2 shows a strong positive association between digital transformation and green product innovation (β = 0.399, p<0.01), providing strong support for H1a. In Model 4, the interaction between digital transformation and incentive regulation has a positive correlation with green product innovation (β = 0.048, p<0.05), supporting H5-1a. Similarly, in Model 6, the interaction between digital transformation and constraint regulation has a positive correlation with green product innovation (β = 0.049, p<0.05), supporting H5-2a. Additionally, in Model 8, the interaction between digital transformation and guidance regulation has optimistic correlation with green competitive advantage (β = 0.057, p<0.01), providing support H5-3a. The research results on the impact of digital transformation on green product innovation are similar to previous studies [43, 55, 56]. The regulatory role played by the government through constraints, incentives, and guidance provides a new perspective for the development of green innovation theory.

**Table 4:** Results of the hierarchical linear regression analysis − Green product innovation.

VARIABLE N**AME	MODEL 1	MODEL 2	MODEL 3	MODEL 4	MODEL 5	MODEL 6	MODEL 7	MODEL 8
**constant**	0.078	-0.118	-0.082	-0.079	-0.094	-0.104	-0.098	-0.116
**Control variables**								
Firm age	-0.069 (-0.081)	-0.002 (-0.002)	-0.007 (-0.008)	-0.006 (-0.007)	-0.004 (-0.004)	-0.002 (-0.002)	-0.010 (-0.012)	-0.007 (-0.009)
Staff number	0.040 (0.071)	0.033 (0.057)	0.029 (0.051)	0.026 (0.046)	0.032 (0.056)	0.030 (0.052)	0.036 (0.063)	0.036 (0.064)
Annual revenue	-0.016 (-0.019)	0.023 (0.027)	0.019 (0.022)	0.015 (0.017)	0.017 (0.020)	0.016 (0.019)	0.014 (0.017)	0.012 (0.013)
Industry	-0.005 (-0.006)	-0.006 (-0.007)	-0.009 (-0.010)	-0.011 (-0.013)	-0.008 (-0.010)	-0.012 (-0.013)	-0.003 (-0.003)	-0.004 (-0.005)
**Main effects**								
Digital transformation		0.399** (0.571)	0.350** (0.501)	0.359** (0.515)	0.318** (0.456)	0.333** (0.477)	0.354** (0.508)	0.369** (0.528)
Incentive regulation			0.132** (0.191)	0.145** (0.209)				
Constraint regulation					0.181** (0.251)	0.193** (0.268)		
Guiding regulation							0.122** (0.171)	0.139** (0.195)
**Interaction effect**								
Digital transformation*Incentive regulation				0.048* (0.098)				
Digital transformation*Constraint regulation						0.049* (0.097)		
Digital transformation*Guiding regulation								0.057** (0.115)
△R^2^	0.012	0.317	0.032	0.009	0.050	0.008	0.025	0.012
△F	1.164	182.099**	18.958**	5.427*	30.802**	5.264*	14.973**	7.125**
AVIF	1.016	1.023	1.071	1.082	1.073	1.122	1.074	1.092

Dependent variable:Green product innovation

Note

** means significant at 1% level

* means significant at 5% level.

Table 5 presents the results with green process innovation as the outcome variable, Models 9–16 show no multicollinearity (AVIF < 5). Model 10 shows a substantial positive correlation between digital transformation and green process innovation (β = 0.493, p<0.01), providing strong support for H1b. In Model 12, the interaction between digital transformation and incentive regulation has a positive correlation with green process innovation (β = 0.049, p<0.05), supporting H5-1b. Likewise, in Model 14, the interaction between digital transformation and constraint regulation has a positive correlation with green process innovation (β = 0.052, p<0.05), supporting H5-2b. Furthermore, in Model 16, the interaction between digital transformation and guidance regulation has a positive correlation with green process innovation (β = 0.059, p<0.01), supporting H5-3b. These research results are similar to previous empirical results, all of which show a significant impact [57, 58]. The research results on the role of digital transformation in green process innovation include the amplifying effect of government regulation on this result, which has become strong evidence for green innovation theory.

**Table 5:** Results of the hierarchical linear regression analysis − Green process innovation.

VARIABLE NAME	MODEL 9	MODEL 10	MODEL 11	MODEL 12	MODEL 13	MODEL 14	MODEL 15	MODEL 16
**constant**	0.05	-0.193	-0.158	-0.156	-0.17	-0.18	-0.173	-0.192
**Control variables**								
Firm age	-0.088 (-0.096)	-0.005 (-0.005)	-0.010 (-0.011)	-0.009 (-0.01)	-0.007 (-0.007)	-0.005 (-0.005)	-0.013 (-0.014)	-0.010 (-0.011)
Staff number	0.059 (0.095)	0.049 (0.080)	0.045 (0.074)	0.043 (0.069)	0.048 (0.078)	0.046 (0.075)	0.052 (0.085)	0.053 (0.086)
Annual revenue	-0.006 (-0.006)	0.043 (0.046)	0.039 (0.041)	0.035 (0.037)	0.037 (0.040)	0.036 (0.039)	0.034 (0.037)	0.032 (0.034)
Industry	-0.005 (-0.005)	-0.006 (-0.006)	-0.008 (-0.009)	-0.011 (-0.012)	-0.008 (-0.009)	-0.012 (-0.012)	-0.003 (-0.003)	-0.004 (-0.004)
**Main effects**								
Digital transformation		0.493** (0.653)	0.447** (0.593)	0.457** (0.606)	0.417** (0.553)	0.432** (0.573)	0.451** (0.597)	0.465** (0.617)
Incentive regulation			0.124** (0.166)	0.137** (0.183)				
Constraint regulation					0.170** (0.218)	0.183** (0.235)		
Guiding regulation							0.116** (0.151)	0.134** (0.174)
**Interaction effect**								
Digital transformation*Incentive regulation				0.049* (0.093)				
Digital transformation*Constraint regulation						0.052* (0.095)		
Digital transformation*Guiding regulation								0.059** (0.110)
△R^2^	0.018	0.415	0.024	0.008	0.038	0.008	0.020	0.011
△F	1.793	281.958**	16.884**	5.670*	27.318**	5.912*	13.752**	7.767**
AVIF	1.016	1.023	1.071	1.082	1.073	1.122	1.074	1.092

Dependent variable: Green process innovation.

Note

** means significant at 1% level

* means significant at 5% level.

Table 6 presents the results with green competitive advantage as the outcome variable, Models 17–22 show no multicollinearity (AVIF < 5). Model 18 shows a notable favorable correlation amidst the digital transformation and green competitive advantage (β = 0.560, p<0.01), offering confirmation for H2. Additionally, Model 19 shows significant correlation (β = 0.700, p<0.01), supporting H3a. Similarly, Model 21 indicates a substantial advantageous association green process innovation and green competitive advantage (β = 0.743, p<0.01), promoting H3b. Based on findings of Model 2, Model 18, and Model 20 (β>0, p<0.01), it can be inferred that hypothesis H4a holds. Similarly, based on the results of Model 10, Model 18, and Model 22 (β>0, p<0.01), it can be inferred that hypothesis H4b also holds [59]. These results are consistent with previous empirical studies [35, 60], demonstrating how digital transformation can enhance green innovation and thus promote green competitive advantage. The research results also contribute to the dynamic capabilities theory, namely that SMEs use digital methods to enhance green innovation through the dynamic capabilities of digital transformation, which enables them to maintain a competitive advantage.

**Table 6:** Results of the hierarchical linear regression analysis − Green competitive advantage.

VARIABLE NAME	MODEL 17	MODEL 18	MODEL 19	MODEL 20	MODEL 21	MODEL 22
**constant**	0.313	0.036	0.258	0.080	0.275	0.124
**Control variables**						
Firm age	-0.073(-0.066)	0.022(0.020)	-0.024(-0.022)	0.022(0.020)	-0.007(-0.007)	0.024(0.022)
Staff number	-0.024(-0.032)	-0.035(-0.047)	-0.052(-0.070)	-0.047(-0.063)	-0.067*(-0.091)	-0.057*(-0.077)
Annual revenue	-0.007(-0.006)	0.048(0.043)	0.004(0.004)	0.040(0.035)	-0.003(-0.002)	0.029(0.025)
Industry	-0.036(-0.031)	-0.037(-0.033)	-0.033(-0.029)	-0.035(-0.031)	-0.033(-0.029)	-0.035(-0.030)
**Main effects**						
Digital transformation		0.560**(0.615)		0.414**(0.455)		0.336**(0.369)
Green product innovation			0.700**(0.536)	0.366**(0.281)		
Green process innovation					0.743**(0.615)	0.454**(0.377)
△R^2^	0.006	0.368	0.284	0.053	0.372	0.080
△F	0.617	226.548**	154.275**	35.413**	230.990**	56.594**
AVIF	1.016	1.023	1.017	1.183	1.020	1.275

Dependent variable:Green competitive advantage

Note

** means significant at 1% level

* means significant at 5% level.

In order to further test the mediation effect, PROCESS v3.3, a plug-in tool for SPSS developed by Hayes, was used for analysis [61]. Table 7 shows that green product innovation and green process innovation have significant mediating effects between digital transformation and green competitive advantage (0.146, [0.096, 0.199]; 0.224, [0.161, 0.318]). Meanwhile, their direct effects’ confidence intervals do not include zero (0.414, [0.329, 0.499]; 0.336, [0.246, 0.426]), indicating green product innovation and green process innovation do not fully facilitate the correlation between digital transformation and green advantage in competition.

**Table 7:** Mediating analysis.

Mediation paths	Direct effect			Indirect effect		
	Effect	LLCI	ULCI	Effect	LLCI	ULCI
Digital transformation→Green product innovation→Green competitive advantage	0.414	0.329	0.499	0.146	0.096	0.199
Digital transformation→Green process innovation→Green competitive advantage	0.336	0.246	0.426	0.224	0.161	0.318

Table 8 lists the outcomes from testing the hypothesis in this study.

**Table 8:** Findings from the testing of research hypotheses.

Hypothesis	Impact path	Result
H1a	Digital transformation is positively related to green product innovation.	Supported
H1b	Digital transformation is positively related to green process innovation.	Supported
H2	Digital transformation is positively related to green competitive advantage.	Supported
H3a	Green product innovation positively related to green competitive advantage.	Supported
H3b	Green process innovation is positively related to green competitive advantage.	Supported
H4a	Green product innovation positively mediates the relationship between digital transformation and green competitive advantage.	Supported
H4b	Green process innovation positively mediates the relationship between digital transformation and green competitive advantage.	Supported
H5-1a	Incentive regulation positively regulates the relationship between digital transformation and green product innovation.	Supported
H5-1b	Incentive regulation positively regulates the relationship between digital transformation and green process innovation.	Supported
H5-2a	Constraint regulation positively regulates the relationship between digital transformation and green product innovation.	Supported
H5-2b	Constraint regulation positively regulates the relationship between digital transformation and green process innovation.	Supported
H5-3a	Guiding regulation positively regulates the relationship between digital transformation and green product innovation.	Supported
H5-3b	Guiding regulation positively regulates the relationship between digital transformation and green process innovation.	Supported

### 4.3 Robustness test

To fully justify the validity of the empirical results, this study adopts two approaches for robustness tests to further verify our main conclusions. First, the robustness test of the research results of this study was conducted by increasing the sample size. The plug-in tool PROCESS v3.3 of SPSS was used to perform bootstrap resampling 5000 times on the original sample, and the 5000 resampled samples were analyzed. The results are shown in Tables 9–11. The test results of the robustness test are consistent with the results shown in Tables 4–6, which strongly supports the main conclusions of the study. Second, a robustness test of the research results of this study was conducted by removing the dummy variables. All the dummy variables in the model were removed, and the original sample was analyzed again. The results are shown in Appendix Tables 12–14. The results of the robustness test are also consistent with the results shown in Tables 4–6. The robustness test results of the above two methods indicate the stability of our empirical results.

**Table 9:** Results of the hierarchical linear regression analysis for robustness check −Green product innovation.

Variables	Model 23				Model 24				Model 25			
	Coeff	BootSE	BootLLCI	BootULCI	Coeff	BootSE	BootLLCI	BootULCI	Coeff	BootSE	BootLLCI	BootULCI
constant	5.110	0.136	4.848	5.378	5.085	0.135	4.817	5.347	5.072	0.138	4.803	5.345
Firm age	-0.006	0.034	-0.074	0.062	-0.002	0.034	-0.065	0.065	-0.007	0.035	-0.074	0.061
Staff number	0.026	0.024	-0.020	0.073	0.030	0.024	-0.016	0.076	0.037	0.024	-0.011	0.084
Annual revenue	0.015	0.034	-0.053	0.082	0.016	0.035	-0.051	0.085	0.012	0.035	-0.055	0.081
Industry	-0.011	0.035	-0.084	0.056	-0.012	0.035	-0.081	0.053	-0.004	0.036	-0.078	0.065
Digital transformation	0.360	0.031	0.297	0.420	0.333	0.033	0.267	0.396	0.369	0.031	0.306	0.429
Incentive regulation	0.145	0.035	0.076	0.213	0.193	0.037	0.122	0.265	0.139	0.036	0.070	0.209
Constraint regulation												
Guiding regulation												
Digital transformation*Incentive regulation	0.048	0.026	0.009	0.112								
Digital transformation*Constraint regulation					0.049	0.026	0.010	0.112				
Digital transformation*Guiding regulation									0.057	0.029	0.015	0.126

**Table 10:** Results of the hierarchical linear regression analysis for robustness check for robustness check −Green process innovation.

Variables	Model 26				Model 27				Model 28			
	Coeff	BootSE	BootLLCI	BootULCI	Coeff	BootSE	BootLLCI	BootULCI	Coeff	BootSE	BootLLCI	BootULCI
constant	5.033	0.138	4.764	5.307	5.009	0.134	4.738	5.268	4.996	0.139	4.721	5.264
Firm age	-0.009	0.034	-0.074	0.057	-0.005	0.033	-0.070	0.061	-0.010	0.034	-0.077	0.057
Staff number	0.043	0.023	-0.002	0.089	0.046	0.023	0.000	0.091	0.053	0.024	0.008	0.100
Annual revenue	0.035	0.035	-0.033	0.103	0.036	0.035	-0.031	0.104	0.032	0.036	-0.037	0.103
Industry	-0.011	0.034	-0.080	0.054	-0.012	0.034	-0.078	0.055	-0.004	0.035	-0.073	0.064
Digital transformation	0.457	0.031	0.395	0.516	0.432	0.031	0.372	0.495	0.465	0.030	0.405	0.523
Incentive regulation	0.137	0.032	0.075	0.200	0.183	0.037	0.110	0.251	0.134	0.035	0.064	0.202
Constraint regulation												
Guiding regulation												
Digital transformation*Incentive regulation	0.049	0.019	0.015	0.093								
Digital transformation*Constraint regulation					0.052	0.021	0.011	0.096				
Digital transformation*Guiding regulation									0.059	0.022	0.022	0.111

**Table 11:** Results of the hierarchical linear regression analysis for robustness check for robustness check−Mediation effect.

Variables	Model 29(Green product innovation as Dependent variable)				Model 30(Green competitive advantage as Dependent variable)				Model 31(Green process innovation as Dependent variable)				Model 32(Green competitive advantage as Dependent variable)			
	Coeff	BootSE	BootLLCI	BootULCI	Coeff	BootSE	BootLLCI	BootULCI	Coeff	BootSE	BootLLCI	BootULCI	Coeff	BootSE	BootLLCI	BootULCI
constant	3.071	0.213	2.652	3.498	1.026	0.323	0.374	1.647	2.524	0.204	2.124	2.924	1.003	0.301	0.401	1.585
Firm age	-0.002	0.036	-0.070	0.068	0.022	0.045	-0.065	0.112	-0.005	0.035	-0.072	0.064	0.024	0.042	-0.061	0.105
Staff number	0.033	0.025	-0.015	0.080	-0.047	0.029	-0.104	0.011	0.049	0.024	0.000	0.094	-0.057	0.028	-0.112	-0.003
Annual revenue	0.023	0.035	-0.046	0.094	0.039	0.044	-0.047	0.125	0.043	0.036	-0.030	0.113	0.028	0.043	-0.057	0.112
Industry	-0.006	0.036	-0.077	0.065	-0.035	0.046	-0.126	0.053	-0.006	0.035	-0.075	0.063	-0.035	0.043	-0.119	0.051
Digital transformation	0.399	0.029	0.341	0.457	0.414	0.046	0.326	0.505	0.493	0.027	0.439	0.546	0.336	0.049	0.241	0.434
Green product innovation					0.367	0.060	0.248	0.480								
Green process innovation													0.455	0.062	0.332	0.578

**Table 12:** Results of the hierarchical linear regression analysis for robustness check − Green product innovation.

**VARIABLE NAME**	**MODEL 33**	**MODEL 34**	**MODEL 35**	**MODEL 36**	**MODEL 37**	**MODEL 38**	**MODEL 39**
constant	-0.001	-0.002	-0.020	-0.002	-0.024	-0.002	-0.022
Digital transformation	0.398**(0.570)	0.349**(0.501)	0.359**(0.515)	0.318**(0.455)	0.332**(0.476)	0.356**(0.510)	0.370**(0.530)
Incentive regulation		0.134**(0.193)	0.146**(0.211)				
Constraint regulation				0.181**(0.252)	0.194**(0.269)		
Guiding regulation						0.121**(0.169)	0.138**(0.194)
Digital transformation*Incentive regulation			0.050*(0.102)				
Digital transformation*Constraint regulation					0.050*(0.099)		
Digital transformation*Guiding regulation							0.057**(0.115)

Dependent variable:Green product innovation

Note

** means significant at 1% level

* means significant at 5% level.

**Table 13:** Results of the hierarchical linear regression analysis for robustness check − Green process innovation.

VARIABLE NAME	MODEL 40	MODEL 41	MODEL 42	MODEL 43	MODEL 44	MODEL 45	MODEL 46
constant	0.003	0.002	-0.017	0.002	-0.022	0.003	-0.018
Digital transformation	0.491**(0.651)	0.445**(0.590)	0.456**(0.604)	0.416**(0.551)	0.431**(0.571)	0.451**(0.598)	0.466**(0.617)
Incentive regulation		0.127**(0.170)	0.140**(0.187)				
Constraint regulation				0.171**(0.220)	0.185**(0.237)		
Guiding regulation						0.115**(0.150)	0.133**(0.173)
Digital transformation*Incentive regulation			0.052*(0.098)				
Digital transformation*Constraint regulation					0.054*(0.098)		
Digital transformation*Guiding regulation							0.059**(0.111)

Dependent variable: Green process innovation.

Note

** means significant at 1% level

* means significant at 5% level.

**Table 14:** Results of the hierarchical linear regression analysis for robustness check− Green competitive advantage.

VARIABLE NAME	MODEL 47	MODEL 48	MODEL 49	MODEL 50	MODEL 51
constant	0.002	0.003	0.003	-0.0002	0.001
Digital transformation	0.553**(0.608)		0.410**(0.450)		0.335**(0.368)
Green product innovation		0.696**(0.534)	0.362**(0.277)		
Green process innovation				0.734**(0.608)	0.445**(0.368)

Dependent variable:Green competitive advantage

Note

** means significant at 1% level

* means significant at 5% level.

## 5. Discussion

Currently, digitization has permeated a plethora of industries [62], and SMEs are grappling with the difficulty of whether and how to undergo digitized transformation to create new sustainable competitive advantages. However, the answer to how digital transformation improves the green competitive advantage of SMEs has not been definitively resolved. Hence, drawing from theory of dynamic capabilities, this study constructed a conceptual framework for investigating exploring the digital transformation, under government regulation, influences SMEs’ green competitive advantage through green innovation.

Firstly, through survey data from 391 SMEs in China, this study research discovered that the adoption of digital transformation can contribute to environmental sustainability green competitive advantage of SMEs. This finding suggests that in the foreseeable future, digital transformation may present a valuable opportunity for businesses to integrate innovation with resources, markets, and value in complex environments, helping SMEs develop innovative activities and transform old, unsustainable competitive advantages into green competitive advantages [63]. Thus, SMEs can consider the process of digitization leading to new way to rapidly build green competitive advantages.

Secondly, our findings indicated that the digital transformation exerts a considerable influence on sustainable technology advancement. In particular, the outcomes demonstrate that the process of digitization has a significant not only accelerates the pace of green innovation for SMEs but also reduces the information search costs of eco-friendly innovation, effectively bridging the information gap, thereby strengthening eagerness of businesses to implement environmentally-friendly creative invention strategies. SMEs undergoing digital transformation can utilize environmentally-conscious innovation to enhance product quality and enhance manufacturing procedures, driving enduring growth of the business through eco-friendly innovation. Previous research on digital transformation has often emphasized the competencies of an organization are generated and enhanced [64, 65], but this study provides improved comprehension of the correlation between digital transformation within green innovation.

Thirdly, it was discovered that SMEs are able to quickly establish more green competitive advantages from digital transformation by means of introducing green product innovation and adopting green process innovation. From one perspective, through drawing parallels [66], SMEs undergoing digital transformation able to strengthen green product innovation strategies and develop their green competitive advantage in new business scenarios. Therefore, green product innovation can provide SMEs with the ability to continuously improve products, linking their digital transformation with their green competitive advantage in the market environment. However, according to Synthesis [67], SMEs have the capability to use green process innovation to improve production processes by replacing old processes with new ones. Previous research has found that companies engaged in the process of acquiring knowledge and developing skills pursuing Radical innovation enables them to sustain their competitive edge [68]. Therefore, as a viable strategy for ongoing success learning in addition to embracing revolutionary innovation initiatives, green process innovation enables SMEs generate strong potential for development of exceptional quality as well as translate benefits of this potential into green competitive advantages.

Fourthly, this study found that government regulation can positively moderate the relationship between digital transformation and green innovation. The research indicates that government incentives, constraints, and guidance regulations can all have a positive impact on digital transformation as well as green innovation. Firstly, the government can create a digital transformation government can implement a governance framework centered on collaborative utilization. Through platform authorization, resources can be gathered and services can be provided to enterprises on the digital transformation platform, disrupting the pattern of fragmented innovation resources, promoting green innovation in enterprises, and using the digital network established to provide alternative competitive advantages for SMEs [69], enabling them to seek support when facing to overcome difficulties in identifying potential clients and business partners [70]. Secondly, the government can promote cooperation and sharing among digital transformation enterprises by strengthening the management mechanisms of digital transformation, creating a transparent, collaborative, and streamlined digital transformation implementing a comprehensive and effective innovation governance framework, and supporting startups and emerging businesses in overcoming these difficulties. This involves enhancing advantageous measures for the construction of cooperative creativity digital transformation networks, expanding digital transformation collaborative innovation, and fully leveraging the regulatory and support roles of government agencies in promoting business digitization and modernization collaborative advancement in successfully advance innovative cooperation projects.

### 5.1 Theoretical implications

The findings from this research have practical significance for theoretical contributions. Firstly, this study enriches the scope of literature research and fills existing gaps in the literature. It supplements the literature on digital transformation research. This study views green innovation as a process rather than an outcome, marking an important conceptual shift.It is rare for existing academic work to focus on the relationship between empirical study of digital switchover and environmental competitive advantage. The results of this study elucidate the logic of green innovation, highlighting the crucial role green innovation plays throughout the process of establishing green competitive advantage in SEMs. SEMs can undergo digital transformation to change their product innovation and process innovation, thereby promoting green competitive advantage. Secondly, it fills in the mechanism of government regulation on SEMs. The results of this research provide insights into how governments can play a key regulatory role throughout the interaction of digital change and environmental innovation. Governments can support enterprises in using digital technology transformation as a catalyst to accelerate the forward movement of green innovation through a series of incentive, constraint, and guidance policies. Observe how the process of digitisation is working in the area of green innovation, bringing about changes and impacts is moderated by government regulation, a conclusion not previously documented in the literature. Secondly, the results of this study systematically analyze based on the perspective of Dynamic Capability Theory, we will deeply analyse how digital technology transformation affects the advantages of green competition and reveal its inherent mechanism of action. Previous research has mainly focused on how large enterprises achieve digital transformation, without exploring issues related to SEMs [71]. This study, through empirical research on SEMs, opens up the internal is a "An internal process that is not easily discernible or transparent" aspect to the digital transformation process that is not easily understood or transparent and competitive position with ecological advantages.Observe how small and medium-sized companies are turning the power of digital transformation into an environmental competitive advantage through green product innovation and green process innovation.This result responds to scholars’ calls to explore the potential mediating effects of SEMs’ competitive advantage in a digital background [72]. The results of this study combine explore how digital change can be combined with the advantages of green competition through a specific lens of internal restructuring, expanding the theoretical boundaries of dynamic capabilities.

### 5.2 Practical implications

The results of this research have led to important insights and implications for both the government and small and SMEs in practical work. From the government’s perspective, firstly, it is essential for the government to improve building a regulatory framework conducive to digital technology transformation through market-based regulatory mechanisms and administrative review processes. In the difficult phase of digital transformation, timely policies are essential to help SMEs overcome challenges. Secondly, the government should continuously enhance environmentally friendly purchasing, environmentally friendly products certification, and green innovation achievement transformation mechanisms to promote the prosperity of the green market, increase the return on activating the green innovation potential of SMEs through environmental innovation. Thirdly, the government should focus on improving governance capabilities, optimizing business and contractual environments, actively guiding SMEs to capitalising on the opportunities presented by digital change, and promoting initiative in digital transformation by implementing preferential measures such as tax reductions, subsidies, etc., to alleviate financial constraints investments in green research and development. From the perspective of SMEs, firstly, SMEs should increase research and development investment, maintain dynamic capabilities, concentrating on innovative research on high-end environmental protection technologies and products and development while implementing digital transformation, and committed to increasing the quantity and quality of high-end environmental innovations. Secondly, SMEs should leverage digital technology resources, explore government financial support and tax relief policies, alleviate Financing Challenges and Promoting Green Innovation Skills. Thirdly, SMEs should adapt to the development trend of digital transformation, seize the opportunity of the new generation of digital technologies and digital platform ecosystems, vigorously promote digital transformation, fully leverage the economies of scale brought by digital transformation, contributing to the long-term development of digital transformation through productivity improvements and cost reductions.

### 5.3 Limitations and future research

The scope and depth of this study is limited that could provide valuable research directions for future studies. Firstly, the relationship between digital transformation and competitive position with ecological advantages was oversimplified in this study. It only focused on the effects of environmental innovations and government regulation, while their relationship may also be influenced by factors such as organizational culture, leadership, and market dynamics. The mechanisms of these variables have not been thoroughly studied, and future research efforts should be directed towards the further study of these variables with a view to achieving a deeper and more comprehensive understanding of their potential mechanisms. Secondly, this study did not focus on analyzing a specific industry. Areas of digital transformation and green innovation in SEMs have their own characteristics in different industries. Although valuable insights have been gained from this study that can guide practical implications, it is important in future in-depth analysis of the role and importance of digital change in various fields and green innovation in different industry contexts. Thirdly, this study only focused on Chinese SEMs. The data collected through empirical surveys may have potential response biases and limitations in the generalizability of the survey results. Different regions may yield different empirical survey results. Comparative analysis between countries may be a valuable exploration direction in future research. Lastly, this study only conducted quantitative analysis of survey data. Since observe how the process of digitisation is working in the area of green innovation, bringing about changes and impacts in SEMs is a long-term process, relying solely on one-time survey data for quantitative studies may not an in-depth analysis of how digital change is comprehensively impacting green competitive advantage. Future research could focus on how digitalisation processes can generate long-term competitive advantages in small and medium-sized businesses, and combine qualitative methods such as gain a fuller understanding of the nuances of digital transformation for small and medium-sized businesses through in-depth interviews or specific case studies.

## Supporting information

S1 Data Green competitive advantage data. (XLSX)
